# TWEAK/Fn14 Signaling Is Required for Liver Regeneration after Partial Hepatectomy in Mice

**DOI:** 10.1371/journal.pone.0083987

**Published:** 2014-01-09

**Authors:** Gamze Karaca, Marzena Swiderska-Syn, Guanhua Xie, Wing-Kin Syn, Leandi Krüger, Mariana Verdelho Machado, Katherine Garman, Steve S. Choi, Gregory A. Michelotti, Linda C. Burkly, Begoña Ochoa, Anna Mae Diehl

**Affiliations:** 1 Division of Gastroenterology, Department of Medicine, Duke University Medical Center, Durham, North Carolina, United States of America; 2 Regeneration and Repair Group, The Institute of Hepatology, Foundation for Liver Research, London, United Kingdom; 3 Department of Hepatology, Barts Health NHS Trust, London, United Kingdom; 4 Departments of Exploratory Science, Discovery Biology, and Validation Biology, Biogen Idec Inc., Cambridge, Massachusetts, United States of America; 5 Department of Physiology, Faculty of Medicine, University of the Basque Country, Bilbao, Spain; University of Basque Country, Spain

## Abstract

**Background & Aims:**

Pro-inflammatory cytokines are important for liver regeneration after partial hepatectomy (PH). Expression of Fibroblast growth factor-inducible 14 (Fn14), the receptor for TNF-like weak inducer of apoptosis (TWEAK), is induced rapidly after PH and remains elevated throughout the period of peak hepatocyte replication. The role of Fn14 in post-PH liver regeneration is uncertain because Fn14 is expressed by liver progenitors and TWEAK-Fn14 interactions stimulate progenitor growth, but replication of mature hepatocytes is thought to drive liver regeneration after PH.

**Methods:**

To clarify the role of TWEAK-Fn14 after PH, we compared post-PH regenerative responses in wild type (WT) mice, Fn14 knockout (KO) mice, TWEAK KO mice, and WT mice treated with anti-TWEAK antibodies.

**Results:**

In WT mice, rare Fn14(+) cells localized with other progenitor markers in peri-portal areas before PH. PH rapidly increased proliferation of Fn14(+) cells; hepatocytic cells that expressed Fn14 and other progenitor markers, such as Lgr5, progressively accumulated from 12–8 h post-PH and then declined to baseline by 96 h. When TWEAK/Fn14 signaling was disrupted, progenitor accumulation, induction of pro-regenerative cytokines, hepatocyte and cholangiocyte proliferation, and over-all survival were inhibited, while post-PH liver damage and bilirubin levels were increased. TWEAK stimulated proliferation and increased Lgr5 expression in cultured liver progenitors, but had no effect on either parameter in cultured primary hepatocytes.

**Conclusions:**

TWEAK-FN14 signaling is necessary for the healthy adult liver to regenerate normally after acute partial hepatectomy.

## Introduction

Healthy adult livers regenerate efficiently after partial hepatectomy (PH). To reconstruct functional hepatic tissue, regeneration requires replacement of all cell types that were lost with the resected liver lobes. Replacement of mature hepatocytes and cholangiocytes is believed to be accomplished by replication of those cell types in the remaining liver. Mechanisms that replenish other cell populations, including progenitors, are unclear.[1]

Progenitors in healthy adult livers localize along canals of Herring (COH), vestiges of the fetal ductal plate that persist around adult liver portal tracts.[2] The COH-associated progenitor population of adult livers includes bipotent progenitors that are capable of differentiating along either the hepatocytic or biliary lineages depending on the demand for replacing the respective mature cell types.[3] This progenitor population expands during chronic liver injury, presumably to keep pace with chronically increased turnover rates of mature liver epithelial cells.[3] 70% of the portal tracts and associated COH are abruptly lost during PH. Thus, PH provides an enormous stimulus to regenerate the hepatic stem/progenitor compartment. Little is known about this process.

Bipotent liver epithelial progenitors express Fn14, a TNF-superfamily receptor for TWEAK (TNF-like weak inducer of apoptosis).[4], [5] TWEAK is a cytokine that is produced by tissue macrophages and other cells during many types of injury.[4], [6] TWEAK-Fn14 interactions promote the growth of Fn14(+) progenitors because knocking down Fn14 or neutralizing TWEAK in mice blocks the expansion of progenitor populations during chronic liver injuries that typically mobilize such cells, while TWEAK treatment promotes the expansion of progenitor populations.[4], [7], [8] The importance of TWEAK/Fn14 signaling in regulating liver progenitor populations was further substantiated by a recent report that bone marrow transplantation generated TWEAK-producing macrophages which stimulated outgrowth of liver progenitors.[6]

Hepatic expression of Fn14 mRNAs increases more than 50 fold within a few hours after PH.[9], [10] The significance of this dramatic induction of Fn14 after PH is uncertain. Herein we evaluate the hypothesis that TWEAK-Fn14 signaling helps to replenish liver progenitor populations in regenerating livers after PH. Various approaches were used to quantify and localize changes in Fn14 expression following PH in healthy adult WT mice, and to map the timing of the Fn14 response to changes in other progenitor markers, proliferative activity in mature liver epithelial cells, recovery of liver mass, and overall survival. Results in WT mice were then compared to these same outcome measures in mice with targeted deletion of Fn14 or TWEAK, and WT mice that were treated with neutralizing anti-TWEAK antibodies. The findings confirm the hypothesis about TWEAK/Fn14 and reconstitution of hepatic progenitor pools, but also reveal that TWEAK/Fn14 signaling is required for otherwise healthy adults to regenerate mature liver epithelial cells, recover healthy liver mass, and survive following acute PH.

## Materials and Methods

### Reagents

Chemicals were obtained from Sigma-Aldrich Corporation (St. Louis, MO) unless stated otherwise.

### Animal Experiments

In total, more than 200 mice were used in these studies. C57BL/6 wild type mice (WT, n = 80), Fn14 knockout (Fn14 KO) mice (n = 60), TWEAK knockout (TWEAK KO) mice (n = 10), and their respective littermate controls (n = 16), were maintained in animal facilities at Duke University. Both KO mice strains were generously provided by Biogen Idec Inc. (Cambridge, MA).[4], [11] Animal surgery and care were approved by the Institutional Animal Care and Use Committee as governed by the National Institute of Health's “Guide for the Care and Use of Laboratory Animals”, Duke University Animal Welfare Assurance Number A3195-01. Animals were sacrificed under general anesthesia and all efforts were made to minimize suffering during surgery.

8 to 10 week old male mice were subjected to 70% partial hepatectomy (PH) according to the method Higgins and Anderson as previously reported.[9] A minimum of 4 mice/group/time point were sacrificed at 12 hours (Fn14 KO mice and controls only), and 24 and 48 hours (all groups) after PH. Depending on availability of knockout mice, studies were repeated to increase sample size. Hence, at least 10 mice/group were sacrificed at the 12, 24 and 48 hour time points. More detailed list of experimental group division was provided in **Table S1**. In some studies, C57BL6 WT mice were injected i.p. with anti-TWEAK antibodies (200 µg/mouse, clone P2D10, Biogen Idec) or a comparable volume of control IgG immediately before PH and daily thereafter. To evaluate treatment effects on proliferative activity, all mice were routinely administered bromo-deoxyuridine (BrdU) intraperitoneally (50 µg/g body weight) 2 hours before sacrifice. Animals were weighed before PH and before sacrifice. Collected liver samples removed at the time of initial PH (0 hour time point) and regenerating liver samples that were harvested at 12, 24, or 48 hours post-PH were weighed and snap frozen in liquid nitrogen or formalin fixed for further analysis.

### Immunohistochemistry and BrdU Labelling

Immunohistochemistry was performed as previously described.[12] Formalin-fixed paraffin-embedded liver tissues were cut into 5 µm thick sections and placed on glass slides. Sections were de-parafinized with xylene and dehydrated with ethanol, then incubated for 10 min in 3% hydrogen peroxide to block endogenous peroxidase. Antigen retrieval was performed by heating in 10 mM sodium citrate buffer (pH 6.0) for 10 min or incubation with pepsin (00-3009; Invitrogen) for 5 min. Sections were blocked in Dako protein block (X9090; Dako) for 15 min and incubated with primary antibodies at 4°C overnight. Primary antibodies used were: keratin19 (Troma III, 1∶400; Hydroma Bank), AFP (A0008, 1∶400; Dako), Fn14 (314102, Biolegend), LGR5 (TA301323, Origene) HRP-conjugated anti-rabbit (K4003; Dako) secondary antibodies were used to visualize target proteins. DAB reagent (K3466; Dako) was applied in the detection procedure. Ki67 and BrdU staining were quantified by counting the numbers of hepatocytes and ductular cells with stained nuclei in ten randomly chosen 20X fields/section/mouse.

### mRNA Quantification by Real Time RT-PCR

Total RNA was isolated using commercial reagents (TRIzol Reagents, Invitrogen). RNA concentration and purity was determined with NanoDrop ND-1000 Spectrophotometer (NanoDrop Technologies, Palo Alto, CA) and samples with a 260/280 nm absorbance ratio of >1.8 were used in subsequent analyses. mRNAs were quantified by QRT-PCR using 25 ng of cDNA, as described.[13] All samples were analyzed in duplicate. Gene expression levels were normalized to the reference gene S9, and fold change was calculated by the 2^−ΔΔCt^ method. Sequences of primers are listed in the **Table S2**.

### Western Blot Analysis

Protein extracts were prepared by homogenization of liver tissue in RIPA buffer (R0278; Sigma) and quantified by Pierce BCA kit. Proteins were visualized by western analysis using the following primary antibodies; keratin 7 (sc-70936, Santa Cruz Biotechnology), alpha-fetoprotein (AFP, A0008, Dako), Fn14 (314102, Biolegend), LGR5 (TA301323, Origene) and β-actin (sc-47778, Santa Cruz Biotechnology).

### Statistical Analysis

Results are expressed as mean ± SEM. Significance was established using the student *t*-test. P<0.05 was considered significant.

## Results

### Partial Hepatectomy Triggers Hepatic Accumulation of Fn14(+) Cells in WT Mice

**Figure 1:**
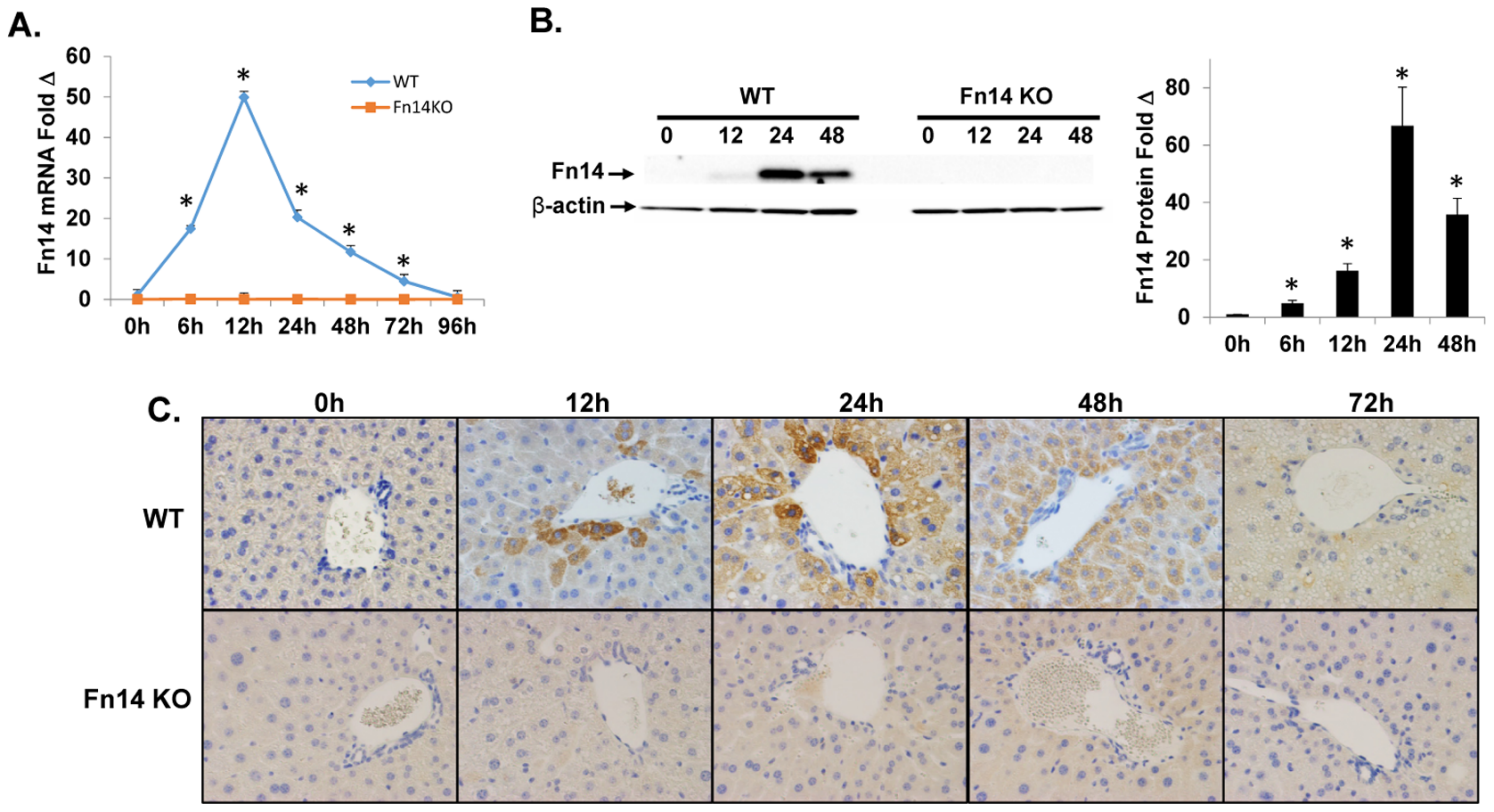
Partial hepatectomy triggers hepatic accumulation of Fn14(+) cells in WT mice. Healthy adult wild type (WT) mice (n = 80) underwent PH. Expression of Fn14 was evaluated at various time points by (A) qRT-PCR analysis, (B) Representative western blot, quantification of protein levels for Fn14 normalized to β-actin and (C) Immunohistochemistry (magnification 20×). To assure reagent specificity, similar analysis was done in mice that were genetically-deficient in Fn14(Fn14 KO). Mean +/− SEM results from all mice (n = 4–5/time point) are graphed relative to baseline, pre-PH (time 0), * p<0.05.

Healthy adult wild type (WT) mice (n = 80) underwent PH. Expression of Fn14 was evaluated at various time points by qRT-PCR analysis, Western blot, and immunohistochemistry. Similar analysis was done in mice that were genetically-deficient in Fn14 (Fn14KO) (n = 60). Consistent with earlier reports,[9], [10] we found that Fn14 mRNA levels were barely detectable at baseline but increased around 50 fold following PH (**Figure 1A**). Transcript induction was maximal at 12 hours post-PH. Western blot analysis demonstrated a comparable induction of Fn14 protein which peaked about 12 hours later and remained 30 fold above basal levels through 48 hours post-PH (**Figure 1B**). Changes in whole liver content of Fn14 protein reflected a dramatic increase in Fn14-expressing cells (**Figure 1C** **and Figure S1**). These cells emerged around portal tracts and then rapidly accumulated in other parts of the liver lobule. Interestingly, however, the morphology of these Fn14(+) cells was not typical of bipotent liver epithelial progenitors, which are generally small cells with an oval nucleus. Rather, the cells that express Fn14 following PH appear hepatocytic. Given the kinetics of the RNA and protein data (**Figure 1A–B**), plus the fact that neither transcripts (**Figure 1A**) nor immunoreactivity (**Figure 1B–C** **and Figure S1**) were demonstrated in Fn14 KO liver, immunohistochemistry evidence for Fn14 expression by hepatocytic cells appears to be valid, and consistent with another report which demonstrated Fn14 expressing hepatocytes in injured human and mouse liver.[5]

### Post-PH growth of Fn14(+) Cells is TWEAK/Fn14-Dependent

**Figure 2:**
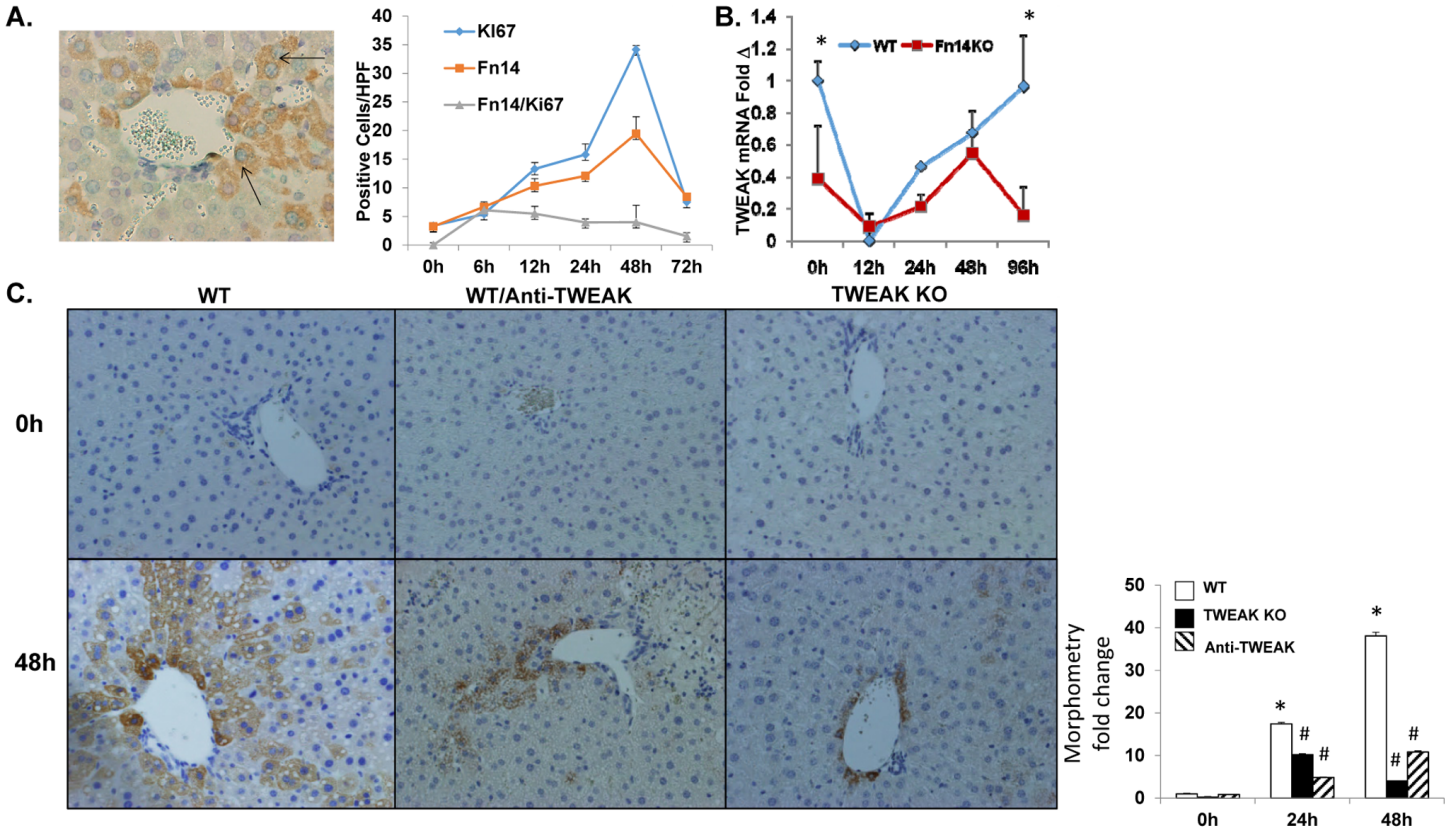
Post- PH growth of Fn14(+) cells is Fn14-dependent and requires TWEAK. (A) Liver sections from WT mice were double-stained for Fn14 (brown) and Ki67 (green), a proliferation marker. Representative image of liver from 6 h after PH is displayed. Arrow indicates double positive cells. Mean +/− SEM numbers of positive cells in 10 random 40× fields/section in 5 mice/group/time point. (B) qRT-PCR analysis of TWEAK mRNA expression in whole liver RNA from WT and Fn14 KO mice at various time points after PH; * p<0.05 vs WT. (C) Fn14 immunohistochemistry and morphometry analysis in WT mice, WT mice treated with Anti-TWEAK antibodies, TWEAK KO mice, and Fn14 KO mice at baseline (0 h) and 48 h after PH. * p<0.05 vs time 0, # p 0.05 vs WT. Representative images are displayed (magnification 20×).

To determine the mechanism for accumulation of these Fn14(+) hepatocytic cells, liver sections from WT mice were double-stained for Fn14 and Ki67, a proliferation marker[14] (**Figure 2A**). Numbers of Fn14/Ki67-double positive cells increased rapidly after PH and were 5 fold greater than baseline by 6 hours post-PH. Proliferative activity in the Fn14(+) compartment gradually declined thereafter, reaching baseline levels by 72 hours after PH. The early peak in Fn14(+) cells preceded increased proliferative activity in Fn14-negative cells, which occurred around 48 hours after PH, the expected time frame for maximal post-PH DNA synthesis in hepatocytes.[1] Interestingly, outgrowth of Fn14(+) cells occurred despite reduced hepatic expression of TWEAK mRNA, which decreased sharply during the initial 12 hours after PH and only returned to basal levels by 96 hours post-PH. TWEAK expression in Fn14 KO mice followed a similar pattern, albeit at generally lower levels, demonstrating that Fn14-expressing cells were not required for TWEAK production after PH (**Figure 2B**). To determine if TWEAK was necessary for accumulation of Fn14(+) cells, immunostaining for Fn14 was done on liver sections from TWEAK KO mice and WT mice treated with either anti-TWEAK antibodies or an irrelevant IgG. Morphometry confirmed striking expansion of the Fn14(+) compartment during the initial 48 hours after PH in WT mice and demonstrated significant attenuation of this response in both TWEAK KO mice and WT mice treated with anti-TWEAK antibodies (**Figure 2C**). Thus, the outgrowth of Fn14(+) cells post-PH is TWEAK-dependent.

### Deletion of Fn14 Inhibits Post-PH Proliferation of Hepatocytes and Cholangiocytes

**Figure 3:**
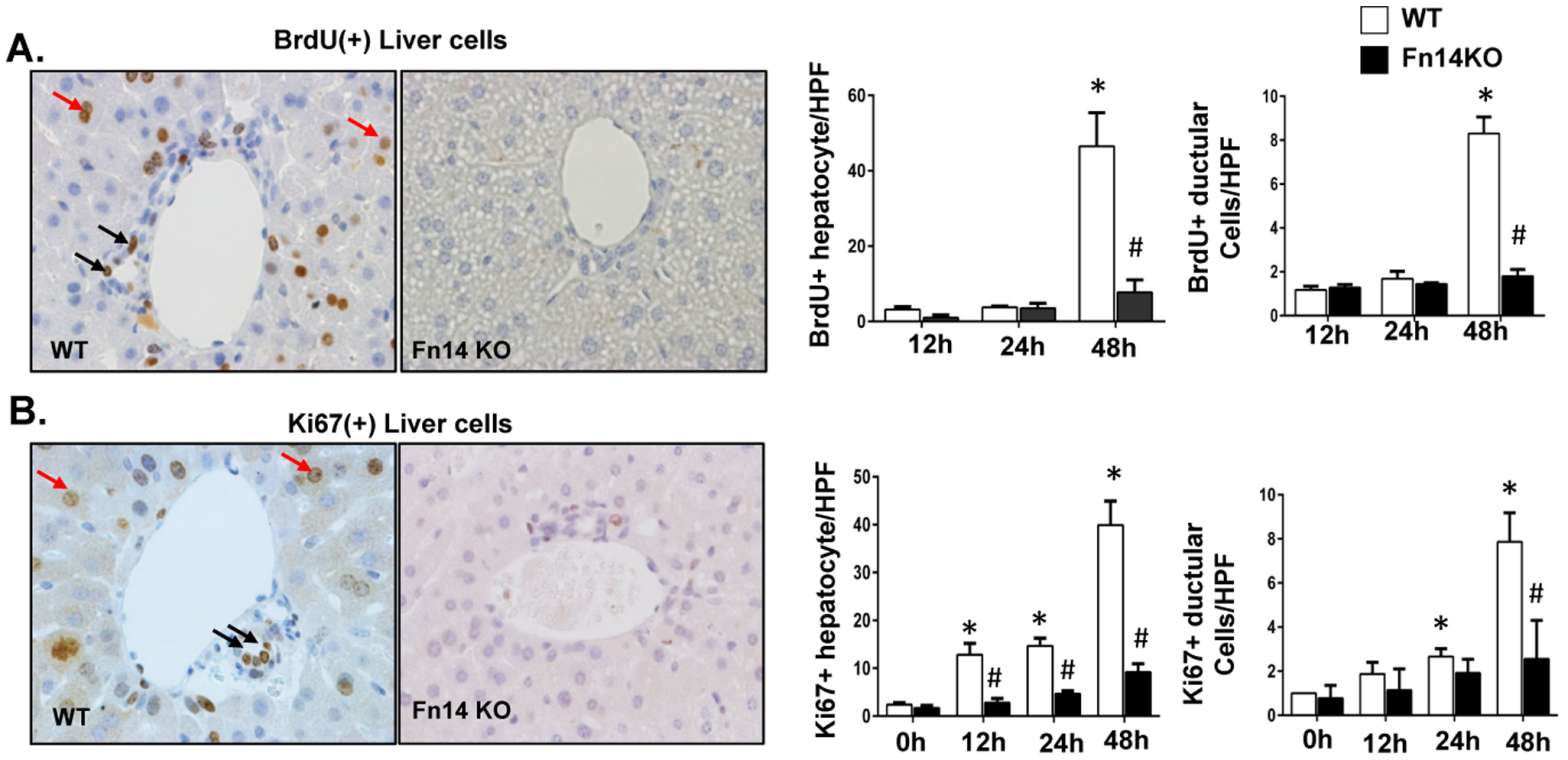
Deletion of Fn14 inhibits post- PH proliferation of hepatocytes and cholangiocytes. Fn14 KO mice and wild type (WT) mice underwent PH. Proliferative activity was evaluated by (A) BrdU incorporation and (B) Ki67 immunostaining. Representative images are displayed (magnification 20×). Arrows indicate Brdu(+) or Ki67(+) cells (red-hepatocyte, black-ductular cells). Labeled hepatocytes/ductular cells were counted in 10 randomly-selected 20× magnification fields/section. Mean +/− SEM results from all mice (n = 3–4/group/time point) are graphed relative to baseline, pre-PH (time 0) in WT group. * p<0.05 vs time 0, # p<0.05 vs WT. Representative images of 48 hours post-PH liver are displayed (magnification 20×).

**Figure 4:**
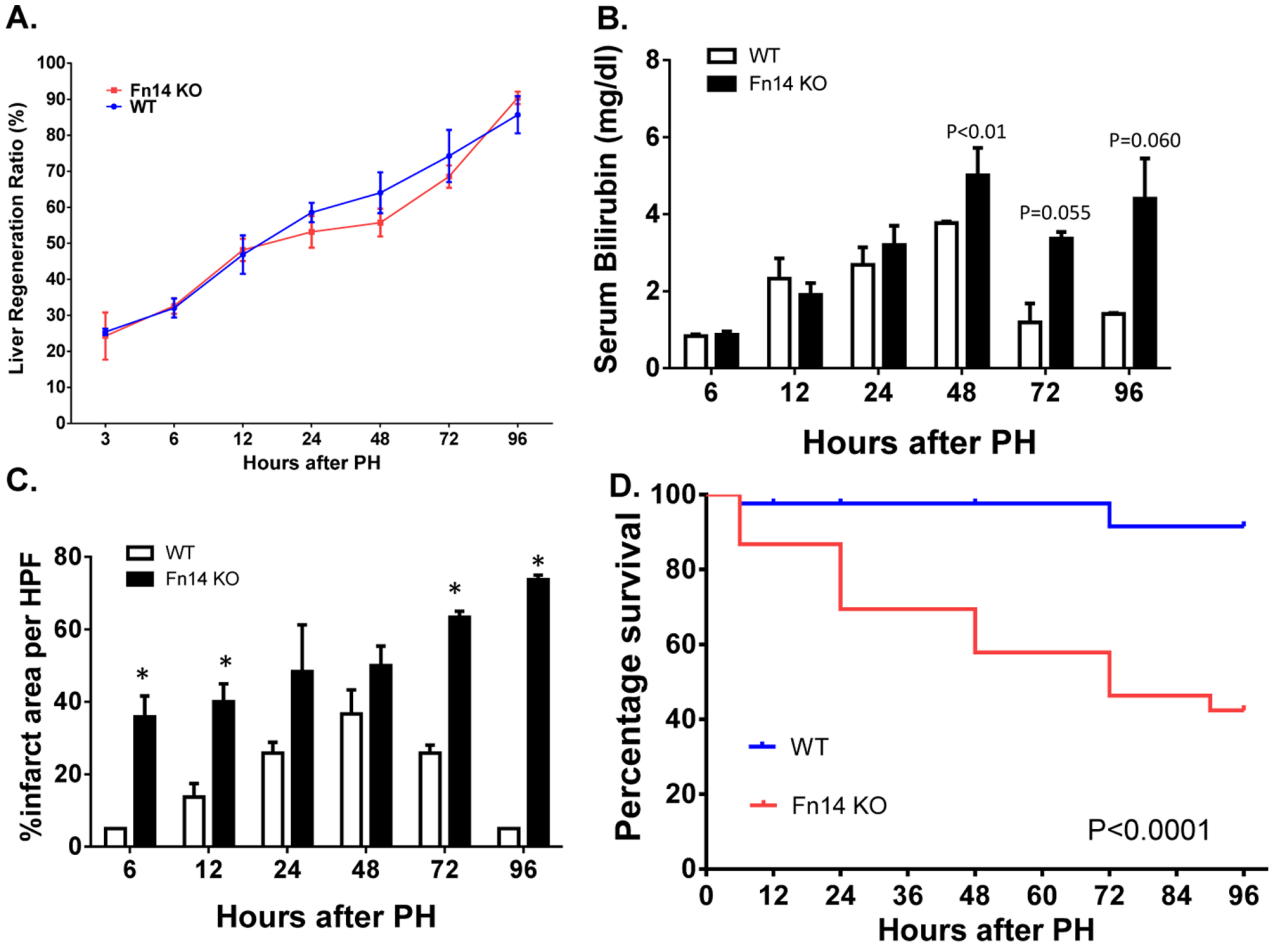
Deletion of Fn14 impairs liver regeneration and survival after PH. Healthy adult wild type (WT) and Fn14 KO mice underwent PH. (A) Liver regeneration ratios (assessed by dividing liver weight at sacrifice by the estimated original liver weight) were compared between WT and Fn14 KO mice at various time points post-PH. (B) Serum bilirubin levels were measured using a Total Bilirubin Test Kit (BIOTRON Diagnostics). (C) Percentage of infarct areas per high-powered field (HPF) were also quantified at in 50 randomly-chosen fields/section. Mean +/− SEM results from 3–4 mice/group/time point are shown. * p<0.01 vs WT. (D) Survival rates were compared between WT and Fn14 KO mice at various time points post-PH. The P values for survival curve are calculated using the log rank test.

Most of the proliferative cells at later time points (e.g., 24–72 hours) post-PH did not express Fn14 (**Figure 2A**). To resolve whether or not Fn14 activity was necessary for this late increase in liver cell proliferation, proliferative activity in hepatocytic and ductular cells was evaluated at various time points in WT and Fn14 KO mice by BrdU incorporation and Ki67 immunostaining. Both approaches demonstrated the expected increase in hepatocyte and ductular cell proliferation in WT mice. In contrast, deletion of Fn14 significantly inhibited the normal post-PH induction of proliferation in both compartments (**Figure 3**), demonstrating that Fn14 is required for optimal regeneration of mature hepatocytes and cholangiocytes following PH. Consistent with these data, net recovery of liver weight in the Fn14 KO group (assessed by regeneration ratio [15]) lagged somewhat behind that of WT mice from 24–72 hours post-PH (**Figure 4A**). Moreover, other outcome measures indicated that loss of Fn14 significantly subverted post-PH recovery of ***functional*** hepatic parenchyma. Rather, areas of infarcted liver increased steadily after PH in Fn14 KO mice (**Figure 4C** **, Figure S2**). This was accompanied by sustained hyperbilirubinemia and progressive mortality. (**Figure 4B,D**). Indeed, by 96 hours after PH, almost 60% of Fn14KO mice were dead compared to about 5% of WT mice, and serum bilirubin levels in surviving Fn14 KO mice were about three fold higher than in WT controls. Hence, loss of Fn14 signaling impairs proper liver regeneration and leads to liver failure after PH.

### Deletion of TWEAK or Inhibition TWEAK Activity with Anti-TWEAK Antibody Inhibit Proliferation of Hepatocytes and Cholangiocytes

**Figure 5:**
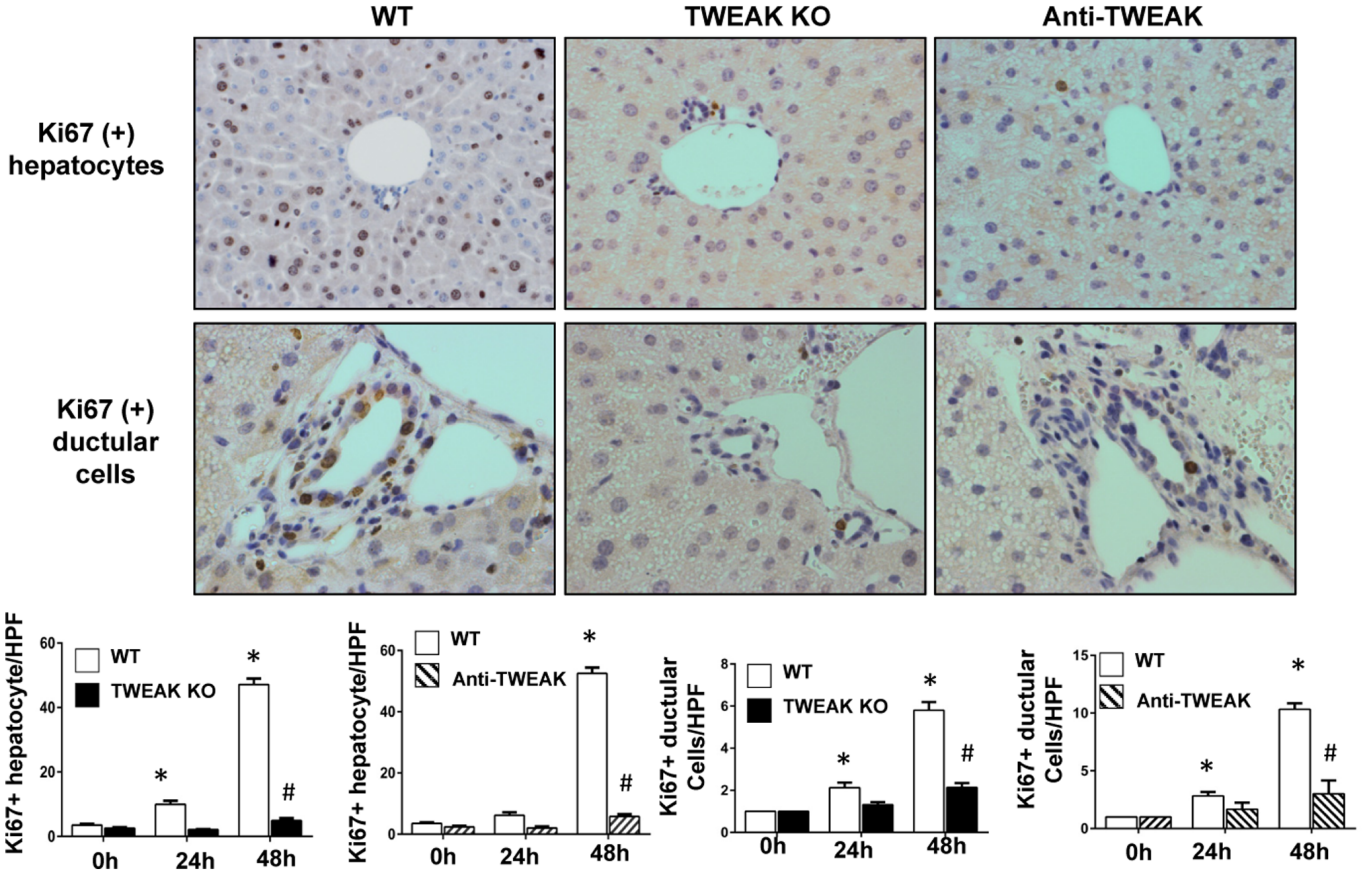
Deletion of TWEAK and inhibition TWEAK activity with anti-TWEAK antibody inhibits proliferation of hepatocytes and cholangiocytes. TWEAK KO mice, WT mice and WT mice that were treated with anti-TWEAK antibodies underwent PH. Ki67 labeled hepatocytes or ductular cells were counted in 10 randomly-selected 20× magnification fields/section. Mean +/− SEM results from all mice (n = 3–4/time point) are graphed relative to baseline, pre-PH (time 0) in WT group. * p<0.05 vs time 0, # p 0.05 vs WT. Representative images of 48 hours post-PH liver are displayed (magnification 20×).

Post-PH outgrowth of Fn14(+) cells is TWEAK dependent (**Figure 2C**) and accumulation of Fn14(+) cells is necessary for optimal regeneration of Fn14-negative hepatocytes and cholangiocytes following PH (**Figure 3**). This suggests that TWEAK activity is also required for increased hepatocyte and ductular cell proliferative activity post-PH. To evaluate this possibility directly, TWEAK KO mice, WT mice treated with anti-TWEAK antibodies, and their respective controls underwent PH and numbers of Ki67-labeled hepatocytes/ductular cells were counted in representative sections of livers harvested at various time points post-PH. The results confirm that inhibiting TWEAK significantly inhibited post-PH proliferation of hepatocytes and cholangiocytes (**Figure 5**), demonstrating that TWEAK-Fn14 signaling is necessary for adult livers to regenerate successfully after PH.

### TWEAK/Fn14 Signaling Stimulates Post-PH Replication of Mature Liver Epithelial Cells via Indirect Mechanisms

Although several earlier studies had demonstrated that TWEAK was unable to stimulate proliferation of healthy mature hepatocytes, evidence that deleting/neutralizing TWEAK or deleting its receptor blocked liver regeneration after PH prompted us to re-examine the direct effects of TWEAK on hepatocyte proliferation. To verify the biological activity of the recombinant TWEAK used for our studies, we tested its effect on a mouse liver progenitor cell line (603B cells) which we had previously characterized and found to exhibit features of liver progenitors.[16] FACS showed that, 603B cells uniformly expressed Fn14 protein (**Figure S3A**). Treating 603B cells with TWEAK increased their expression of AFP[17] and LGR5 [3], markers of hepatocytic progenitors (**Figure S3C**). Doses of TWEAK as low as 1 ng/ml also stimulated their growth (**Figure S3B**). However, as others reported,[4], [7] we found that recombinant TWEAK was unable to stimulate proliferation of primary hepatocytes isolated from the livers of healthy adult mice even at doses as high as 10 ng/ml (**Figure S3B**), or when used in conjunction with IL6, a putative hepatotrophic cytokine (data not shown). Consistent with the inability of TWEAK to directly impact hepatocyte proliferative activity, neither qRT-PCR analysis (data not shown), nor flow cytometry (**Figure S3A**) were able to demonstrate expression of Fn14 by primary hepatocytes isolated from healthy adult mice livers. The aggregate data, therefore, demonstrate that after PH, TWEAK directly stimulates an early wave of replication in Fn14(+) progenitors, but promotes the subsequent proliferative surge of mature liver epithelial cells via indirect mechanisms.

**Figure 6:**
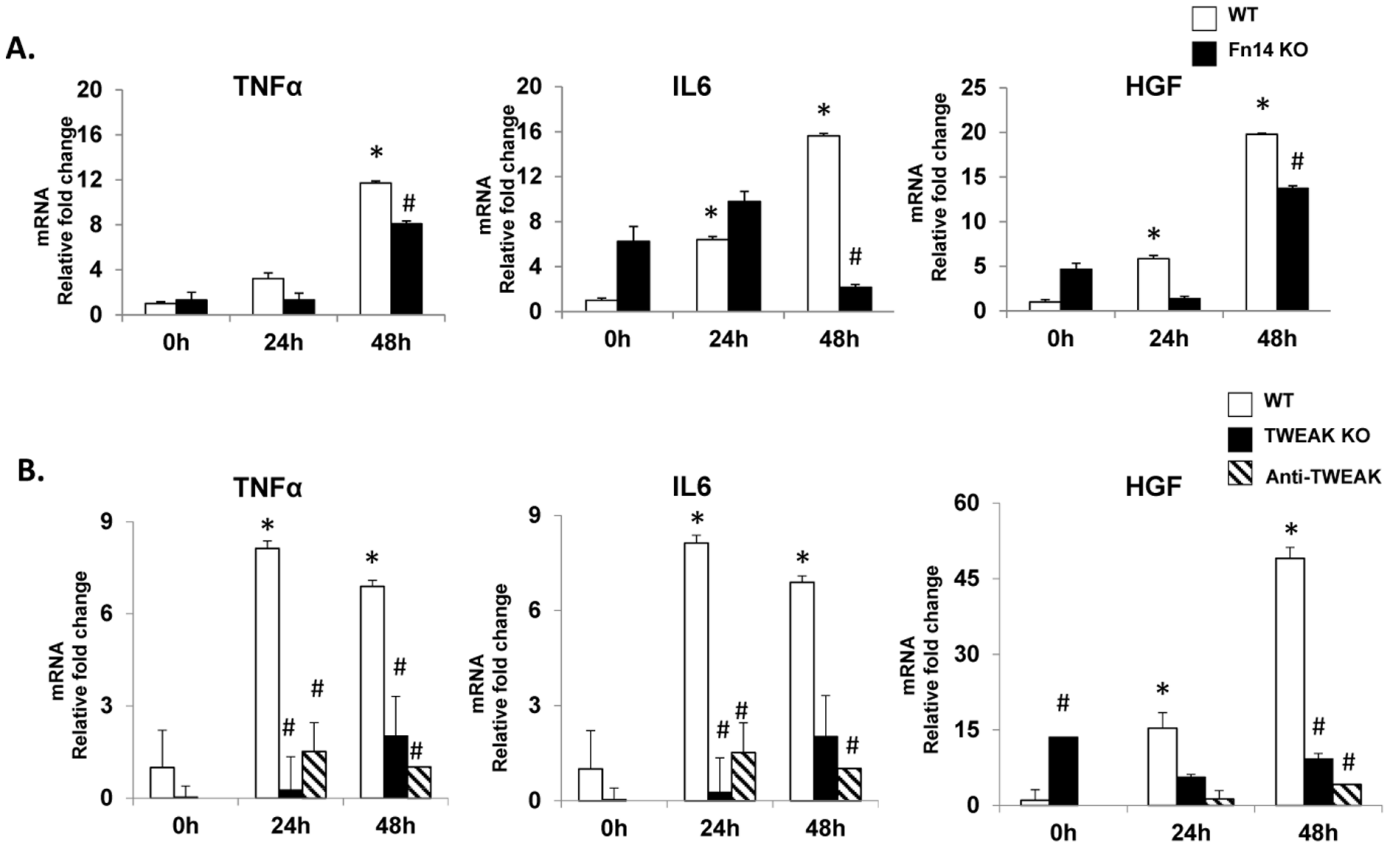
Inhibiting TWEAK/Fn14 signaling attenuates the induction of mitogens associated with liver regeneration. QRT-PCR analysis of whole liver expression of TNFα, IL6 and HGF mRNA in (A) WT and Fn14 KO mice (B) WT, TWEAK KO and WT mice treated with Anti-TWEAK antibodies after PH. Mean +/− SEM results from all mice (n = 3–4/time point) are graphed relative to baseline, pre-PH (time 0) in WT group. * p<0.05 vs time 0, # p<0.05 vs WT.

One such mechanism might involve TWEAK/Fn14-related induction of other liver growth factors. Cytokines and growth factors that increase rapidly after PH function as co-mitogens to promote hepatocyte proliferation after PH. In this regard, tumor necrosis factor (TNF) alpha, interleukin (IL)-6 and hepatocyte growth factor (HGF) are particularly noteworthy because blocking the activities of any of these factors is sufficient to inhibit induction of hepatocyte DNA synthesis and liver regeneration following PH.[1] Quantitative RT-PCR analysis of whole liver RNA from Fn14 KO mice, TWEAK KO mice, WT mice treated with anti-TWEAK antibodies, and their respective wild-type controls demonstrated that inhibiting TWEAK-Fn14 signaling inhibited normal post-PH induction of each of these factors (**Figure 6**). Therefore, loss of TWEAK/Fn14-regulated growth factors likely contributed to the inhibited proliferation of mature-appearing hepatocytes and ductular cells that occurred after PH when TWEAK/Fn14 signaling was disrupted. Additional mechanisms might also be involved, however, including reduced availability of Fn14-positive progenitors to differentiate into more mature, Fn14-negative liver cells that proliferate in response to these other mitogens at later time points after PH. This possibility merits consideration because PH abruptly removes not only 70% of mature liver epithelial cells, but also 70% of the COH-associated progenitor cells.

### Deletion of Fn14 Inhibits Accumulation of Progenitor Cells after PH

**Figure 7:**
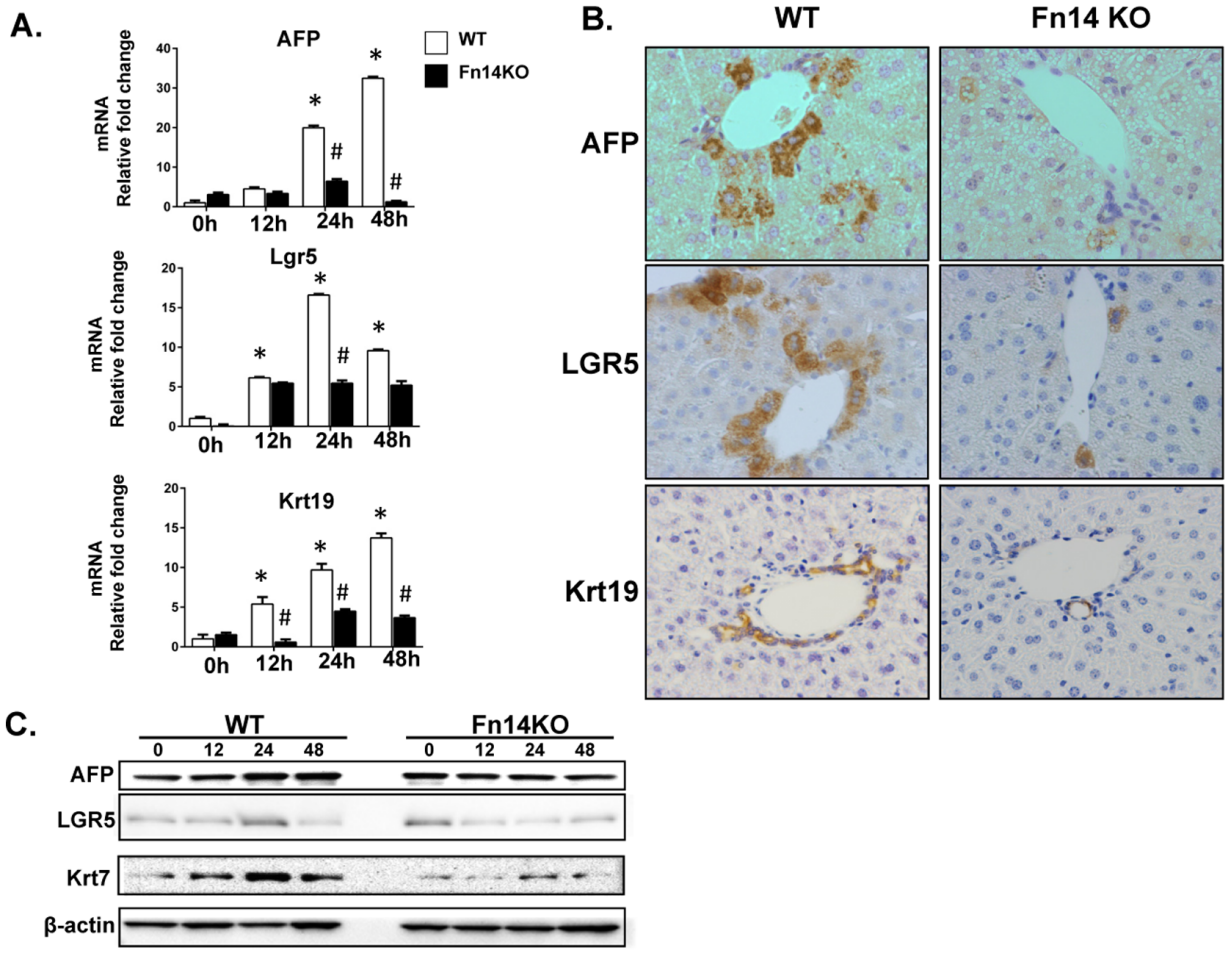
Deletion of Fn14 inhibits accumulation of progenitor cells after PH. Fn14 KO mice and wild type (WT) mice underwent PH. Expression of progenitor markers was evaluated at various time points by (A) qRT-PCR analysis, (B) Immunohistochemistry and (C) Western blot. Mean +/− SEM results from all mice (n = 5/time point) are graphed relative to baseline, pre-PH (time 0) in WT group, * p<0.05 vs time 0, # p<0.05 vs WT. Representative images of 48 hours post-PH liver are displayed (magnification 40×).

**Figure 8:**
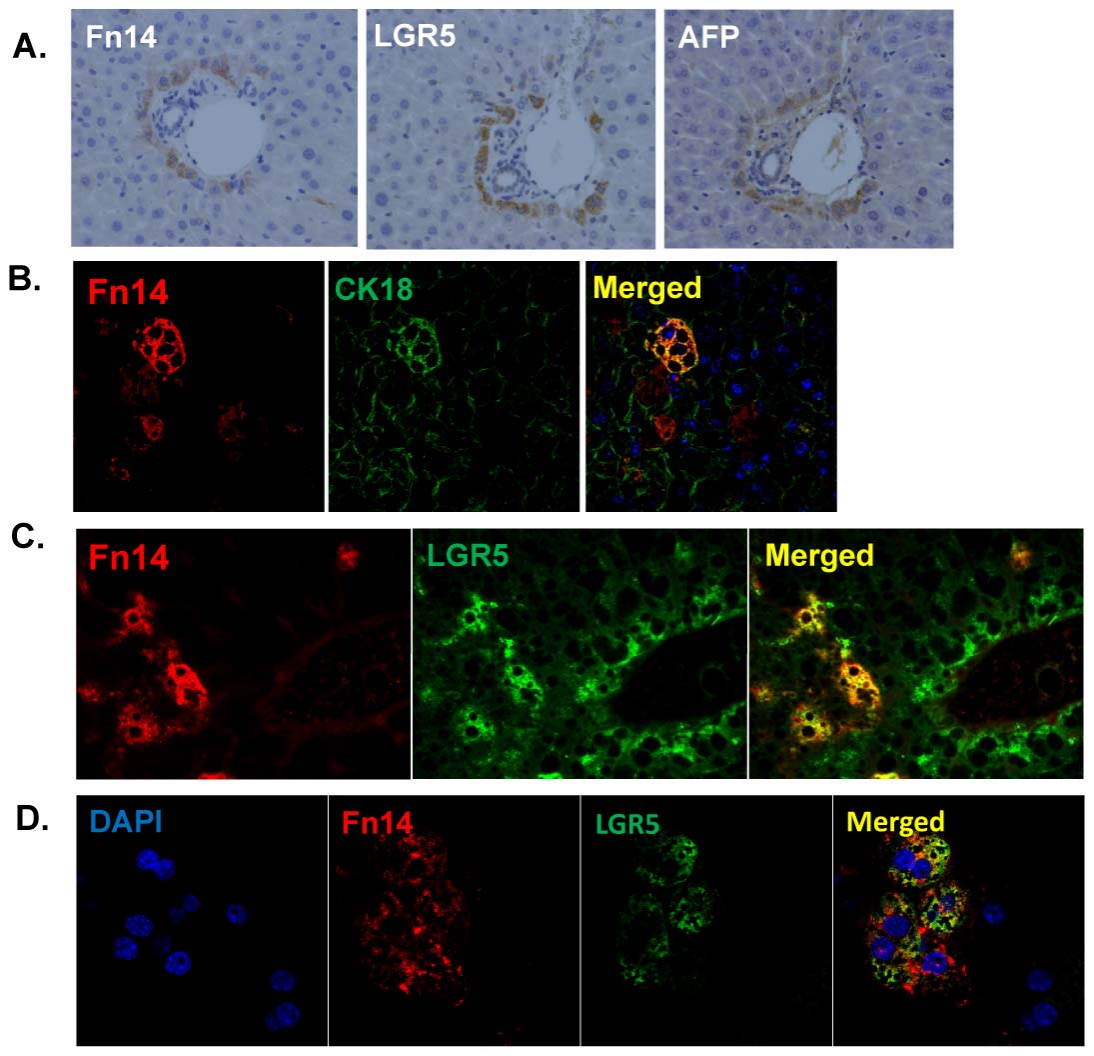
Liver Fn14(+) cells co-localize with progenitor cells. (A) Serial section staining for Fn14 and progenitor markers (AFP and LGR5) in WT mice 48 hours after PH (magnification 40×). (B) Confocal images of Fn14 and CK18 co-staining in WT mice liver 24 hours after PH (magnification 40×). Yellow indicates co-localization of these two markers. (C) Double immunofluorescence for Fn14 and LGR5 in WT mice liver 48 hours after PH (magnification 20×). Yellow indicates co-localization of these two markers. (C) Confocal images of Fn14 and LGR5 co-stained primary hepatocytes isolated from WT mice 24 hours after PH (magnification 63×).

Consistent with the concept that PH provides a strong stimulus to regenerate the progenitor compartment, we observed increased expression of Fn14 in hepatocytic cells near COH within a few hours after PH (**Figure 1C** **, Figure S1**). To evaluate the effects of PH on expression of other progenitor markers, and determine whether or not loss of Fn14 impacted this, expression of other progenitor markers was evaluated by immunohistochemistry, qRT-PCR analysis, and Western blot. At baseline, cells expressing AFP (a marker of hepatocytic progenitors) or LGR5 (an endodermal progenitor marker) were rarely observed, and expression of Krt19 (a marker for immature and mature ductular cells [18]) was restricted to intralobular bile ducts in both WT and Fn14KO mice (**Figure S4**). In WT mice following PH, hepatocytic cells expressing AFP and LGR5 emerged in peri-portal areas and gradually accumulated in other regions of the hepatic parenchyma, while Krt19 staining remained largely restricted to ductular structures around portal tracts. However, the post-PH accumulation of AFP, LGR5 and Krt19-expressing cells was severely attenuated in Fn14 KO mice (**Figure 7B** **and Figure S4**). These changes were substantiated by PCR and Western blot analysis, demonstrating significant post-PH increases in mRNA and protein expression of AFP, LGR5, Krt19, and Krt7 (another liver progenitor marker [18]) in WT mice, but consistent suppression of these responses in Fn14 KO mice (**Figure 7A,C**). Hence, Fn14 is required for progenitor cells to accumulate normally after PH. Moreover, careful inspection of serially-stained sections demonstrated that Fn14(+) cells were closely localized with LGR5(+) cells and AFP(+) cells at each time point, but distinct from cells that expressed Krt19 (**Figure 7** **,** **Figure 8A**), suggesting that Fn14 marks hepatocytic progenitors after PH. To confirm this finding, we performed double immunofluorescence staining of Fn14 and CK18 (an epithelial cytokeratin that is expressed by both mature hepatocytes and hepatocyte progenitors) on liver sections from WT mice after PH. In addition, double staining of Fn14 and LGR5 was assessed on both liver sections and isolated hepatocytes from WT mice after PH (**Figure 8B–D**). Fn14 co-localized with CK18 and LGR5 in a subpopulation of hepatocytes, providing strong evidence that Fn14 is expressed by hepatocytic progenitors and demonstrating that the hepatocyte compartment becomes enriched with such progenitors a day or so before Fn14-negative hepatocytes begin to proliferate.

### TWEAK Is Required for Optimal Regeneration of Liver Progenitors after PH

**Figure 9:**
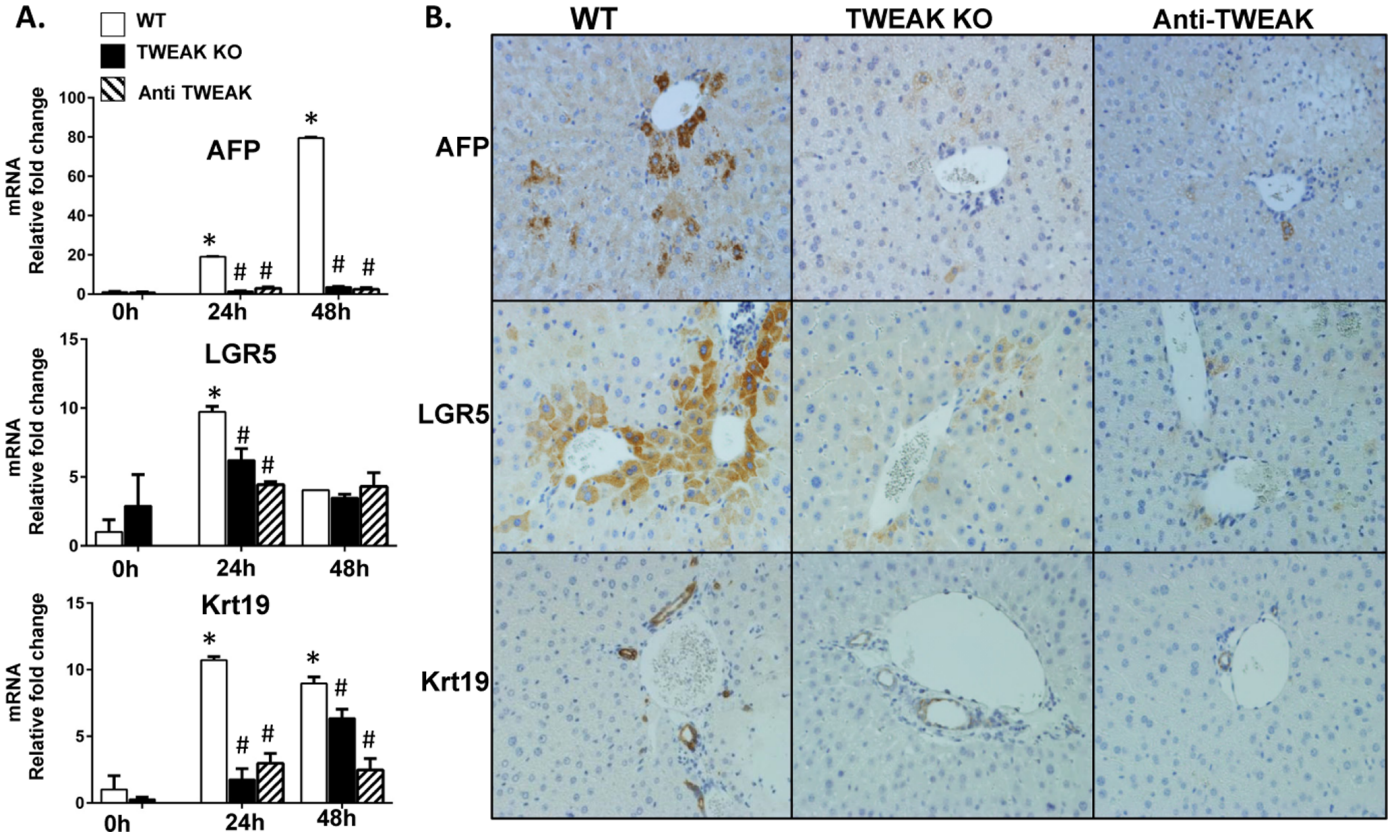
TWEAK is required for optimal liver regeneration after PH. TWEAK KO mice, WT mice and WT mice that were treated with anti-TWEAK antibodies underwent PH. Expression of progenitor markers was evaluated at various time points by (A) qRT-PCR analysis and (B) Immunohistochemistry. Mean +/− SEM results from all mice (n = 5/time point) are graphed relative to baseline, pre-PH (time 0) in WT group, * p<0.05 vs time 0, # p<0.05 vs WT. Representative images of 48 hours post-PH liver are displayed (magnification 20×).

Having shown that Fn14 is required for expansion of progenitor populations following PH (**Figure 7**), we wished to determine if TWEAK is also necessary for this aspect of post-PH regeneration. Therefore, we compared progenitor responses to PH in WT mice, TWEAK KO mice, and WT mice that were treated with anti-TWEAK antibodies. QRT-PCR and immunohistochemistry demonstrated that inhibiting TWEAK significantly inhibited post-PH induction of markers for hepatocytic progenitors (AFP and LGR5), and ductular cell precursors (Krt19) (**Figure 9**). Hence, TWEAK and its receptor, Fn14 are necessary for regeneration of both mature hepatocytes and cholangiocytes (**Figure 3** **and** **5**), and their respective precursors (**Figure 7** **and** **9**), following PH.

## Discussion

Our results show, for the first time, that TWEAK/Fn14 signaling is required for optimal induction of various processes that are necessary for livers to regenerate after PH, including activation of hepatocyte/cholangiocyte proliferation, induction of key mitogenic factors, and accumulation of liver progenitors. These new observations extend earlier evidence that PH leads to rapid and strong up-regulation of hepatic Fn14 mRNA expression.[9], [10]

Fn14 mRNAs are known to be expressed by liver cell lines that exhibit features of immature, relatively undifferentiated cells, and TWEAK promotes the proliferation of such cells in culture.[4], [7] In addition, disrupting Fn14 or blocking TWEAK in animal models of liver regeneration that depend upon liver progenitors severely inhibits liver regeneration.[4], [7], [8] The present study provides novel evidence that TWEAK/Fn14 signaling is also necessary to expand liver progenitor populations and regenerate the liver following PH. Thus, it is reasonable to conclude that liver regeneration after PH also depends upon TWEAK-responsive Fn14(+) progenitors.

Evidence that progenitors mediate post-PH regeneration was unexpected because current dogma posits that the process is driven mainly by the replication of residual mature liver epithelial cells in the remnant liver.[19] Therefore, we used FACS analysis to determine if mature hepatocytes express Fn14 and tested whether or not TWEAK was able to increase their proliferative activity directly. We were unable to demonstrate Fn14 on the surface of primary hepatocytes isolated from healthy adult livers, and TWEAK was unable to induce these cells to proliferate. Our results are in accordance with earlier publications which also failed to demonstrate appreciable expression of Fn14 in mature hepatocytes in healthy adult livers.[4], [7] Moreover, that earlier work demonstrated that hepatocyte proliferation was not increased in TWEAK-transgenic mice although such animals strongly expressed TWEAK in hepatocytes and maintained relatively high serum levels of TWEAK.[4] Thus, disruption of TWEAK/Fn14 signaling in mature hepatocytes does not explain why we observed inhibited proliferation of such cells in Fn14 KO and TWEAK KO mice.

Mice that were deficient in TWEAK or FN14 were noted to have defective induction of certain pro-regenerative cytokines (e.g., TNFα and IL6) and hepatocyte mitogens, such as HGF. Thus, it is conceivable that reduced availability of hepato-trophic factors contributed to the inhibited regenerative responses to PH that occurred when TWEAK/Fn14 signaling was disrupted. Another hypothesis that merits consideration is that some replicating hepatocytes in post-PH livers may be the recent progeny of Fn14(+) TWEAK-sensitive liver progenitors. These more mature liver cells no longer express Fn14 and hence, have lost the ability to replicate in response to TWEAK. Our data support this possibility. We found that TWEAK directly stimulated proliferation of Fn14(+) liver progenitors and increased their expression of AFP and LGR5 *in vitro*. We also demonstrated co-localization of Fn14 and these other progenitor markers in regenerating livers after PH, and confirmed co-expression of Fn14 and LGR5 in hepatocytic cells isolated from 24 hours post-PH livers. Recent lineage tracing studies that tracked the fate of LGR5(+) cells in regenerating livers after acute CCl_4_-induced injury demonstrated that some of the repopulating hepatocytes were derived from LGR5(+) progenitors.[3] Our data suggest that the same process might occur after PH.

Moreover, our findings provide novel evidence that TWEAK/Fn14 signaling regulates accumulation of LGR5(+) cells during liver regeneration. In WT mice after PH, we showed that the increased proliferative activity and accumulation of Fn14(+)/LGR5(+) cells was TWEAK-dependent, and resulted in enrichment of the hepatocytic compartment with Fn14(+) progenitor cells before proliferative activity increased in hepatocytes that did not express progenitor markers. In livers that lacked Fn14 (or which had reduced TWEAK activity), LGR5(+) liver progenitors failed to accumulate normally after PH; proliferation of hepatocytes was severely inhibited; and the livers were unable to regenerate normally, resulting in progressive liver damage, liver dysfunction, and mortality rather than recovery. Based on the aggregate results, therefore, our working hypothesis is that many of the hepatocytes that normally repopulate adult livers after PH are the progeny of TWEAK-responsive progenitors that co-express Fn14 and LGR5. Testing this hypothesis requires lineage tracing approaches. Thus, future experiments that genetically label Fn14(+) cells and follow the fate of these cells after PH are needed to resolve this issue.

In summary, the current results prove that TWEAK/Fn14 signaling is required for the liver to regenerate normally following PH. The data demonstrate that key aspects of the regenerative response, including induction of hepatocyte/cholangiocyte proliferation, accumulation of hepato-trophic factors, and expansion of hepatic progenitor populations depend upon signaling that is activated when TWEAK engages its receptor, Fn14. These insights explain earlier observations that Fn14 expression is up-regulated dramatically after PH and improve understanding about mechanisms that control regeneration of adult livers after injury.

## Supporting Information

Figure S1 Partial hepatectomy triggers hepatic accumulation of Fn14(+) cells in WT mice. Healthy adult wild type (WT) mice and Fn14 KO mice underwent PH. Expression of Fn14 was evaluated at various time points by Immunohistochemistry (magnification 10×).
(TIF)

Figure S2 Deletion of Fn14 impairs liver regeneration after PH. Healthy adult wild type (WT) mice and Fn14 KO mice underwent PH. Representative H&E staining of liver sections from WT and Fn14 KO mice at different time points after PH were displayed (magnification 10×). Note Fn14 KO mice livers have more infarct areas than WT mice.
(TIF)

Figure S3 TWEAK promotes proliferation and differentiation of mouse bipotent epithelial liver progenitor cells (603B) but not primary mouse hepaotcyte. (A) FACS analysis of Fn14 protein expression by 603B and freshly isolated wild type primary mouse hepatocyte (mHep). (B) 603B cells and primary mouse hepatocyte (mHep) were cultured with 1 ng/ml recombinant TWEAK for 24 hours. 10 µM BrdU was added to the plates and cells were incubated for 2 hours. Cell proliferation were assessed by BrdU Cell Proliferation Assay Kit. * p<0.05. (C) 603B cells were treated with recombinant Tweak as described in (B) for 24 and 48 hours. mRNA was isolated and changes in α-Fetoprotein (AFP) and LGR5 gene expression were analyzed by qRT-PCR. Results are graphed relative to control at each time point.
(TIF)

Figure S4 Deletion of Fn14 inhibits progenitor response after PH. Wild type (WT) and Fn14 KO mice underwent PH. Expression of progenitor markers (AFP, LGR5 and Krt19) was evaluated at 0 and 48 hours after PH by Immunohistochemistry. Representative images are displayed (magnification 10×).
(TIF)

Table S1 Detailed usage of mice for PH.
(DOCX)

Table S2 Sequence of mouse primers used in experiments.
(DOCX)
