# Supplementary figures and images for: TWEAK/Fn14 Signaling Is Required for Liver Regeneration after Partial Hepatectomy in Mice
Source: PLoS One. 2014 Jan 9;9(1):e83987. doi: 10.1371/journal.pone.0083987 (PMC3886973; doi:10.1371/journal.pone.0083987)

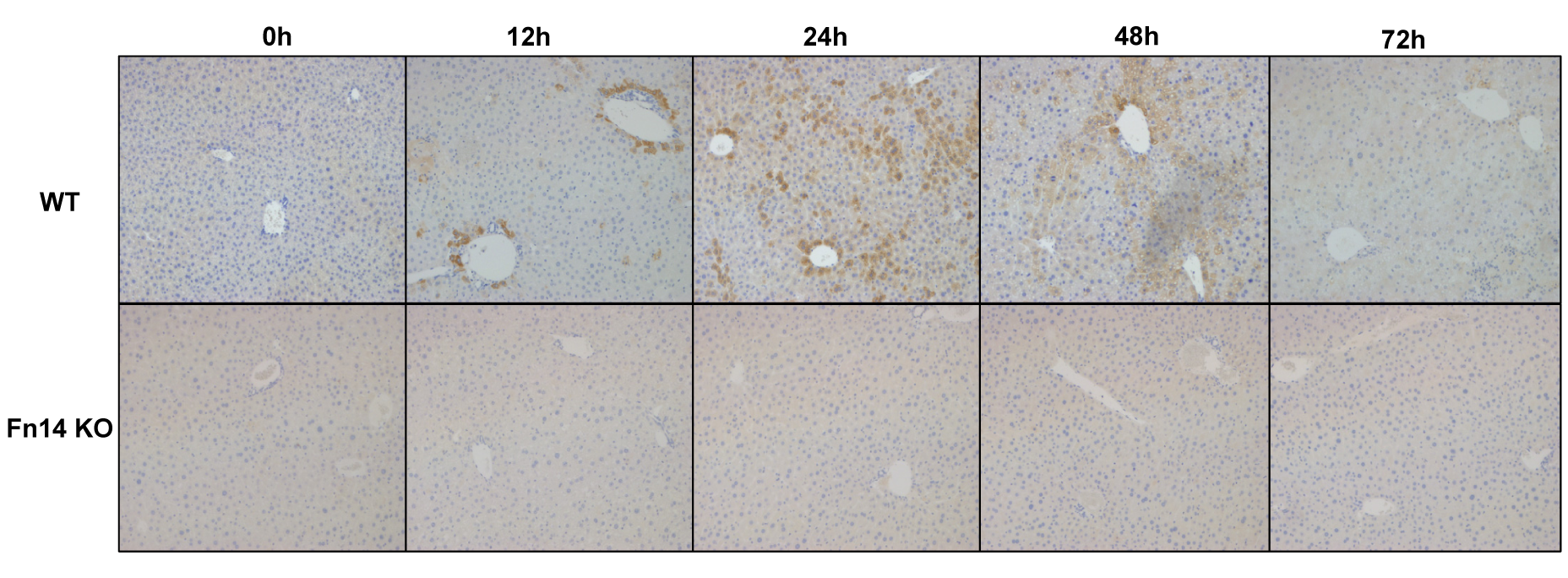

Supplement: Figure S1 — Partial hepatectomy triggers hepatic accumulation of Fn14(+) cells in WT mice. Healthy adult wild type (WT) mice and Fn14 KO mice underwent PH. Expression of Fn14 was evaluated at various time points by Immunohistochemistry (magnification 10×). (TIF) [file pone.0083987.s001.tif]

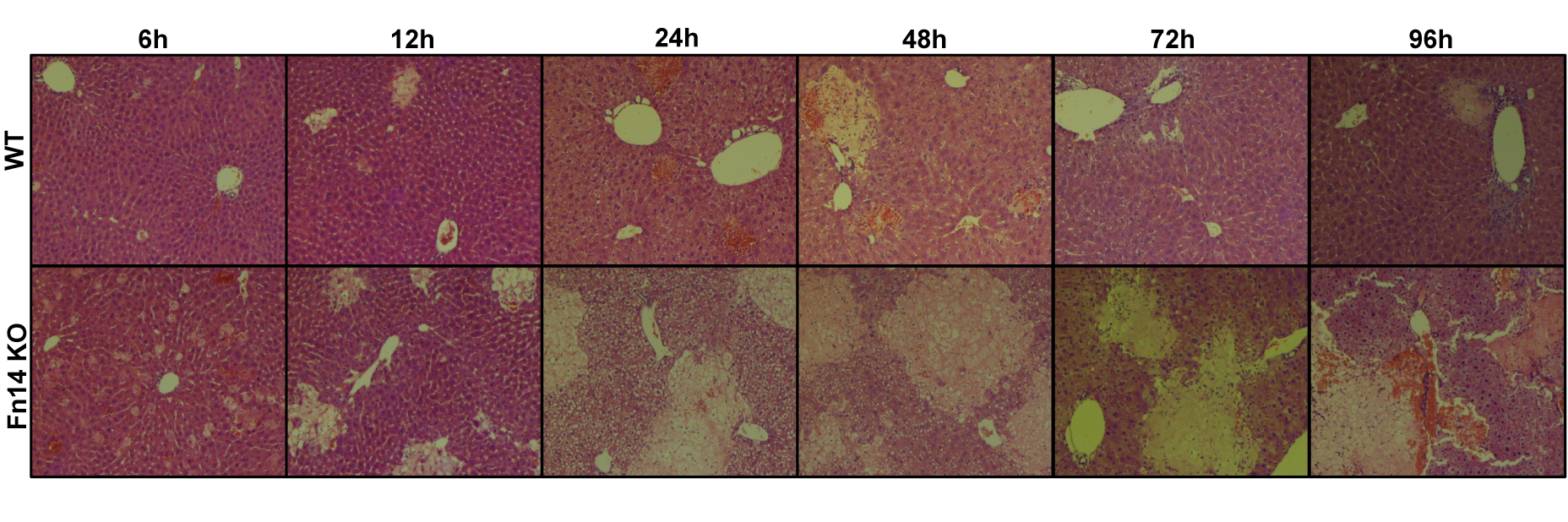

Supplement: Figure S2 — Deletion of Fn14 impairs liver regeneration after PH. Healthy adult wild type (WT) mice and Fn14 KO mice underwent PH. Representative H&E staining of liver sections from WT and Fn14 KO mice at different time points after PH were displayed (magnification 10×). Note Fn14 KO mice livers have more infarct areas than WT mice. (TIF) [file pone.0083987.s002.tif]

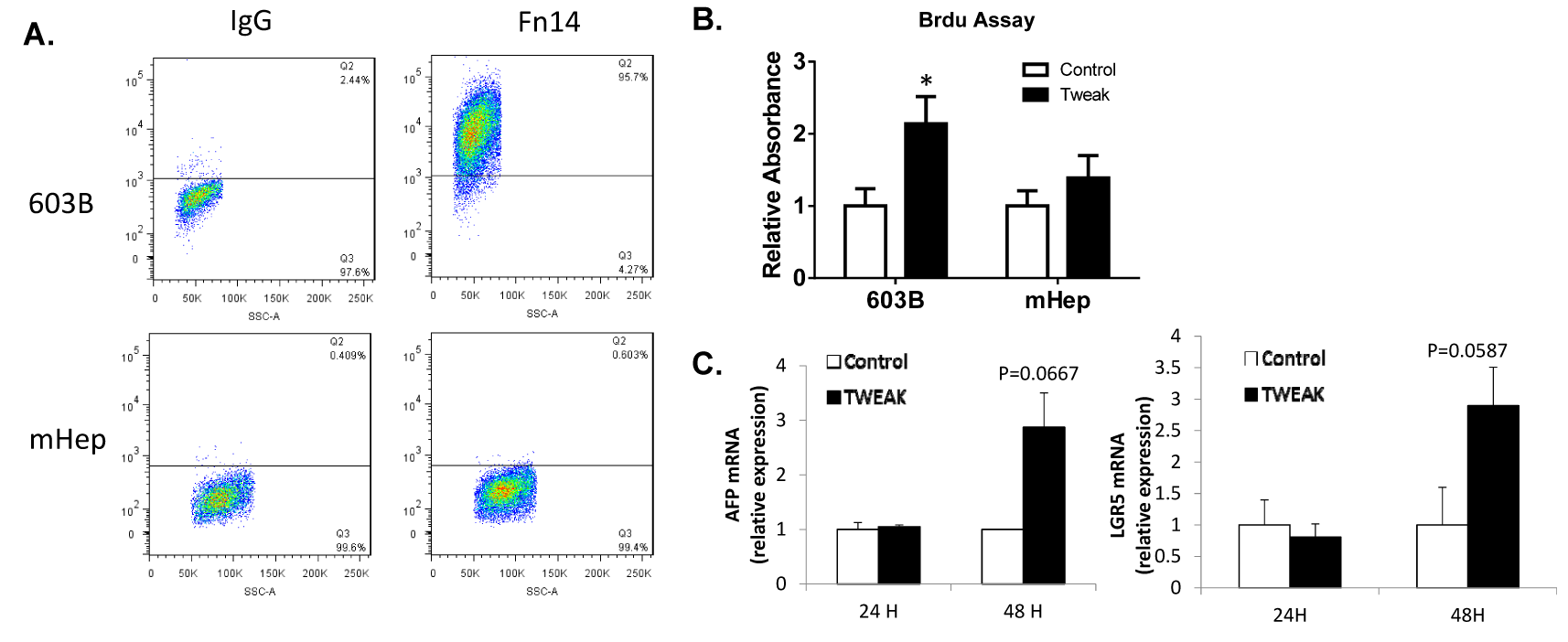

Supplement: Figure S3 — TWEAK promotes proliferation and differentiation of mouse bipotent epithelial liver progenitor cells (603B) but not primary mouse hepaotcyte. (A) FACS analysis of Fn14 protein expression by 603B and freshly isolated wild type primary mouse hepatocyte (mHep). (B) 603B cells and primary mouse hepatocyte (mHep) were cultured with 1 ng/ml recombinant TWEAK for 24 hours. 10 µM BrdU was added to the plates and cells were incubated for 2 hours. Cell proliferation were assessed by BrdU Cell Proliferation Assay Kit. * p<0.05. (C) 603B cells were treated with recombinant Tweak as described in (B) for 24 and 48 hours. mRNA was isolated and changes in α-Fetoprotein (AFP) and LGR5 gene expression were analyzed by qRT-PCR. Results are graphed relative to control at each time point. (TIF) [file pone.0083987.s003.tif]

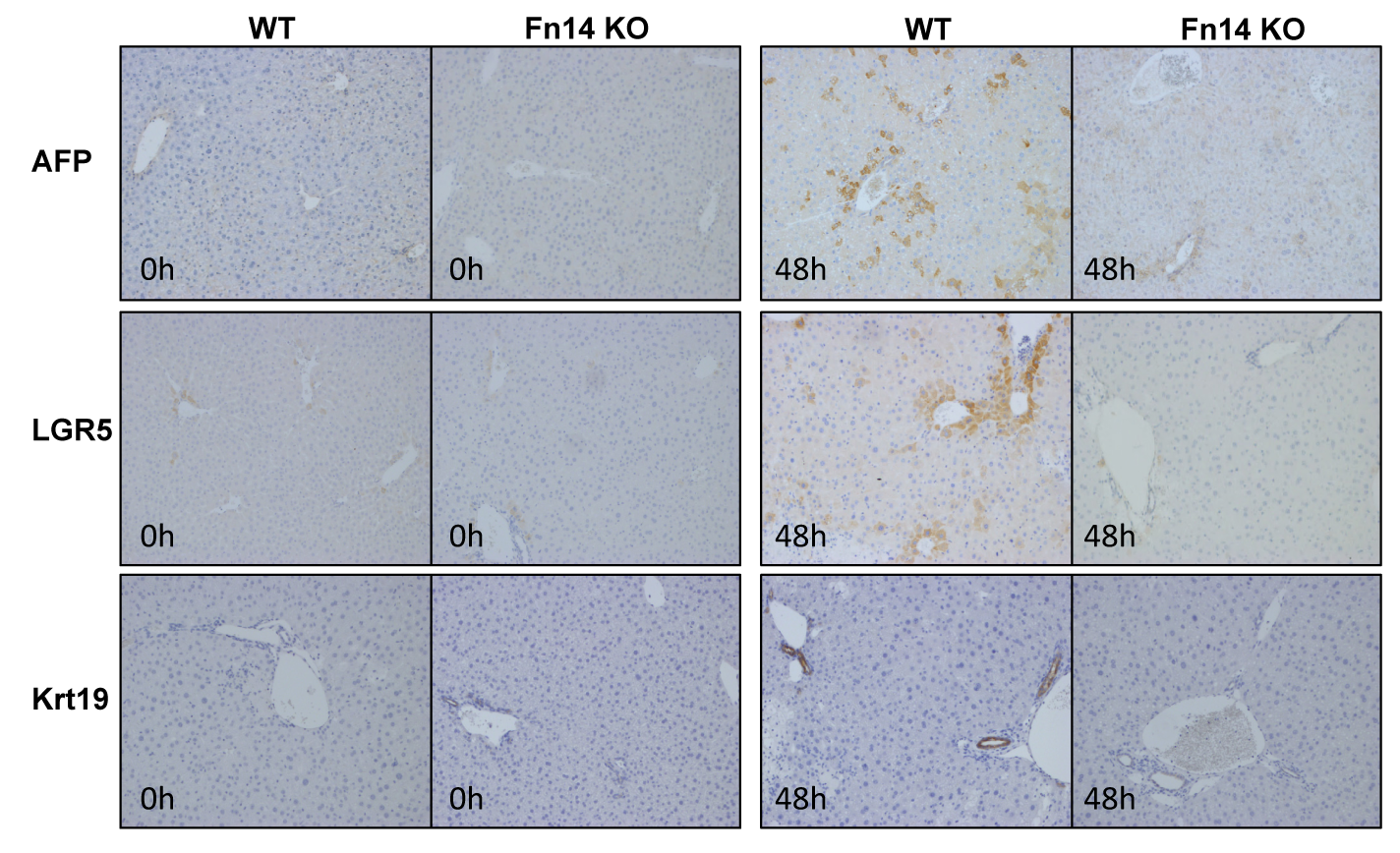

Supplement: Figure S4 — Deletion of Fn14 inhibits progenitor response after PH. Wild type (WT) and Fn14 KO mice underwent PH. Expression of progenitor markers (AFP, LGR5 and Krt19) was evaluated at 0 and 48 hours after PH by Immunohistochemistry. Representative images are displayed (magnification 10×). (TIF) [file pone.0083987.s004.tif]
