# Supplementary material for: TWEAK/Fn14 Signaling Is Required for Liver Regeneration after Partial Hepatectomy in Mice
Source: PLoS One. 2014 Jan 9;9(1):e83987. doi: 10.1371/journal.pone.0083987 (PMC3886973; doi:10.1371/journal.pone.0083987)
Supplement: Table S1 — Detailed usage of mice for PH. (DOCX) [file pone.0083987.s005.docx]

**Table S1: Detailed usage of mice for PH**

| Group | 3h | 6h | 12h | 24h | 48h | 72h | 96h | Total number |
| --- | --- | --- | --- | --- | --- | --- | --- | --- |
| WT | 3 | 7 | 7 | 18 | 18 | 13 | 14 | 80 |
| Fn14 KO | 3 | 6 | 6 | 12 | 12 | 10 | 11 | 60 |
| Tweak KO | 0 | 0 | 0 | 5 | 5 | 0 | 0 | 10 |
| Litter mate control for Tweak KO | 0 | 0 | 0 | 8 | 8 | 0 | 0 | 16 |
