# Supplementary material for: TWEAK/Fn14 Signaling Is Required for Liver Regeneration after Partial Hepatectomy in Mice
Source: PLoS One. 2014 Jan 9;9(1):e83987. doi: 10.1371/journal.pone.0083987 (PMC3886973; doi:10.1371/journal.pone.0083987)
Supplement: Table S2 — Sequence of mouse primers used in experiments. (DOCX) [file pone.0083987.s006.docx]

**Table S2: Sequence of mouse primers used in experiments**

| **Product** | **Forward sequence** | **Reverse Sequence** |
| --- | --- | --- |
| Krt19 | GTGAAGATCCGCGACTGGT | AGGCGAGCATTGTCAATCTG |
| Krt7 | TAGAGTCCAGCATCGCAGAG | CACAGGTCCCATTCCGTC |
| AFP | CCGAGGAGGAAGTGAAACAAA | GGCTTTCTAAACACCCATCG |
| S9 | GACTCCGGAACAAACGTGAGGT | CTTCATCTTGCCCTCGTCCA |
| TNFα | TCGTAGCAAACCACCAAGTG | AGATAGCAAATCGGCTGACG |
| IL6 | AAAGCCAGAGTCCTTCAGAGAGATACAG | ATGAATTGGATGGTCTTGGTCCTTAG |
| Fn14 | GTGTTGGGATTCGGCTTGGT | CCAGGCAGAAGTCGCTGTG |
| TWEAK | ACAACTAGTCCGGCCTCGAAGA | CCCAGCCCTGATGACCGTAA |
| LGR5 | GGATTCCACAGCAACAACATCAG | CGAGGCACCATTCAAAGTCAGTG |
| HGF | ACTGAAGCTTGCTCGACCTG | GTGTAGCCCCAGCCGTAAAT |
